# *Reynoutria japonica* Houtt. Transformed Hairy Root Cultures as an Effective Platform for Producing Phenolic Compounds with Strong Bactericidal Properties

**DOI:** 10.3390/ijms26010362

**Published:** 2025-01-03

**Authors:** Wojciech Makowski, Aleksandra Królicka, Krzysztof Hinc, Agnieszka Szopa, Paweł Kubica, Julia Sroka, Barbara Tokarz, Krzysztof Michał Tokarz

**Affiliations:** 1 Department of Botany, Physiology and Plant Protection, Faculty of Biotechnology and Horticulture, University of Agriculture in Kraków, 29 Listopada 54, 31-425 Kraków, Poland; barbara.tokarz@urk.edu.pl (B.T.); krzysztof.tokarz@urk.edu.pl (K.M.T.); 2 Laboratory of Biologically Active Compounds, Intercollegiate Faculty of Biotechnology UG and MUG, University of Gdańsk, Abrahama 58, 80-307 Gdańsk, Poland; aleksandra.krolicka@ug.edu.pl (A.K.); julia.sroka@phdstud.ug.edu.pl (J.S.); 3 Division of Molecular Bacteriology, Medical University of Gdańsk, Dębinki 1, 80-211 Gdańsk, Poland; krzysztof.hinc@gumed.edu.pl; 4 Department of Medicinal Plant and Mushroom Biotechnology, Collegium Medicum, Jagiellonian University, Medyczna 9, 30-688 Kraków, Poland; a.szopa@uj.edu.pl (A.S.); p.kubica@uj.edu.pl (P.K.)

**Keywords:** Japanese knotweed, *Rhizobium rhizogenes*, flawan-3-ols, antioxidant activity, *Staphylococcus aureus*

## Abstract

*Reynoutria japonica* Houtt. is the source of various phenolic compounds: phenolic acids, flawan-3-ols, and stilbenes, with a broad range of biological activity. The rhizome (underground organ of these plants) is abundant in secondary metabolites but, in natural conditions, may accumulate various toxic substances (such as heavy metals) from the soil. The principal objective of this research was to produce transformed cultures of *R. japonica* hairy roots that would serve as a valuable source of phenolic compounds, independent of environmental resources. The transformation was performed using a variety of wild strains of *Rhizobium rhizogenes* bacteria, of which only strain A4 (ATCC 31798) proved effective. The molecular characterization of transformed clones was performed using PCR. The biometric parameters (growth index and dry weight content), phenolic compounds accumulation (DAD-HPLC), antioxidant capacity (DPPH, CUPRAC), and bactericidal properties against *Staphylococcus aureus* with various sensitivity to antibiotics were evaluated. Two obtained transformed clones (RJ 9 and 30) exhibited the incorporation of the entire bacterial T-DNA into genomic DNA, while clones RJ 10 and 11 demonstrated only the presence of the LT-DNA sequence. The results demonstrated an increase in flawan-3-ols (catechins) accumulation in hairy root tissue relative to non-transformed (NT) plants. Moreover, hairy roots exhibited enhanced antioxidant activity and bactericidal properties compared with NT roots and NT shoots, respectively.

## 1. Introduction

The genetic transformation of medical plants with wild strains of *Rhizobium rhizogenes* bacteria represents a robust biotechnology tool that can be employed to obtain cultures of hairy roots [1]. In this process, the natural ability of the Gram-negative *R. rhizogenes* bacteria to transfer and incorporate bacterial T-DNA into the plant DNA is used. As a result, bacterial oncogenes are introduced into the genomic DNA of plants. Their expression leads to changes in the hormonal balance of plants, which results in increased auxin synthesis. Therefore, the so-called hairy root syndrome occurs, changing the phenotype of the transformed organism [2]. Transformed hairy root cultures are characterized by genetic and phenotype stability, fast growth without the addition of plant growth regulators, and a high accumulation of secondary metabolites [3]. This is due to the fact that bacterial oncogenes modulate physiological processes related to plant response to stress. Therefore, in transformed tissues of hairy roots, they can act as endogenous elicitors of plant secondary metabolites [4,5,6]. This enables the utilization of hairy root cultures as a source of valuable secondary metabolites with a wide spectrum of biological activity. Furthermore, they can serve as a model in studies on the synthesis pathways of secondary metabolites and as a source of knowledge about gene transfer between the bacterial and plant kingdoms [7].

In recent years, there has been an increasing demand for plant raw materials rich in biologically active compounds [7]. This is associated with the rising prevalence of lifestyle-related illnesses and the growing resistance of bacterial pathogens to commercially available antibiotics [8]. A number of secondary plant metabolites, including phenolic compounds, demonstrate strong antioxidant, anti-inflammatory, and antibacterial effects [9]. In the natural environment, phenolic compounds represent an integral element of the plant immune system. They enable plants to defend themselves against unfavorable environmental conditions [10]. Moreover, studies have revealed that they can be successfully employed in the treatment of bacterial infections, acting in synergy with antibiotics [11]. Consequently, the pursuit of novel, efficient, and natural sources of phenolic compounds represents a significant research endeavor.

This study focused on obtaining transformed hairy root cultures of *Reynoutria japonica* Houtt. (syn. *Fallopia japonica* (Houtt.) Ronse Decr.; *Polygonum cuspidatum* Sieb. and Zucc.), commonly known as Japanese knotweed. This plant belongs to the Polygonaceae family, and it is a rich source of phenolic derivatives [12]. The plant has long been exploited in traditional Asian natural medicine [13], and its rhizome is listed in the *Chinese Pharmacopoeia* as a medicinal raw material with a wide range of applications [14]. Accordingly, the utilization of genetic transformation with wild strains of *R. rhizogenes* bacteria to generate hairy root cultures of *R. japonica* offers a promising avenue for the acquisition of underground organ cultures enriched with phenolic compounds.

Due to its status as a highly invasive species, Japanese knotweed cannot be cultivated in field conditions in Europe and the United States [15]. Conversely, plants that occur in their natural habitats cannot be utilized as a source of secondary metabolites. There has been evidence that Japanese knotweed, which is most frequently observed in degraded areas, accumulates toxic substances within its tissues, including heavy metals [16,17,18]. Consequently, in our preceding research, we procured tissue cultures of Japanese knotweed plants, which permitted their cultivation in controlled conditions, regardless of environmental resources. Studies on plants grown in vitro have indicated elevated concentrations of secondary metabolites in their roots compared to plants grown in ex vitro conditions. This finding has established a robust foundation for research on genetic transformation [19].

Therefore, the main objectives of this study were to (1) obtain transformed hairy roots of Japanese knotweed plants, (2) examine the biomass growth of transformed clones, (3) qualitatively and quantitatively analyze the phytochemical composition of extracts from transformed clones, (4) examine the productivity of synthesis of phenolic compounds in transformed plants, and (5) determine the biologically active properties of extracts from cultures of hairy roots of Japanese knotweed. We hypothesized that transformed hairy roots may be characterized by constant and effective growth with a simultaneous increase in the level of secondary metabolites. Increased synthesis of phenolic compounds may lead to increased antioxidant and bactericidal potential of extracts from transformed plants.

## 2. Results

### 2.1. Obtaining Transformed Hairy Root Clones

**Figure 1:**
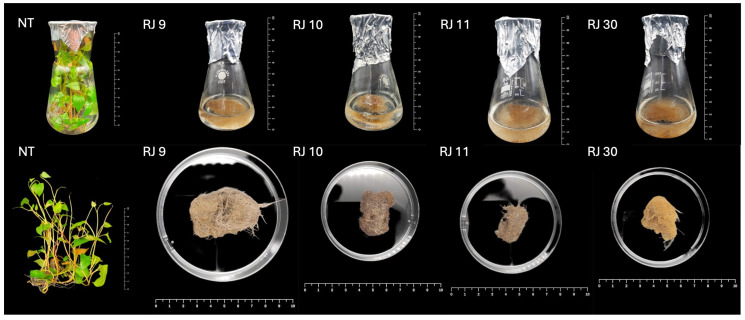
Non-transformed (NT) plants and transformed hairy root cultures (clones: RJ 9, RJ 10, RJ 11, and RJ 30) of *Reynoutria japonica* after 5 weeks of cultivation.

The inoculation of *R. japonica* leaves explants was performed using three wild strains of *R. rhizogenes* bacteria. Only strain A4 (ATCC 31798) resulted in the appearance of axenic hairy roots at the explants with an effectiveness of 64%. Hairy root production was observed after 4 weeks after inoculation. Teratomas (transformed shoots) production was not observed in hairy root cultures. Based on the preliminary results from the growth observations and the screening for the phenolic compound accumulation in the obtained hairy root clones, four transformed hairy root clones of *R. japonica* were taken for further analysis: RJ 9, RJ 10, RJ 11, and RJ 30 (Figure 1).

### 2.2. Molecular Analysis

**Figure 2:**
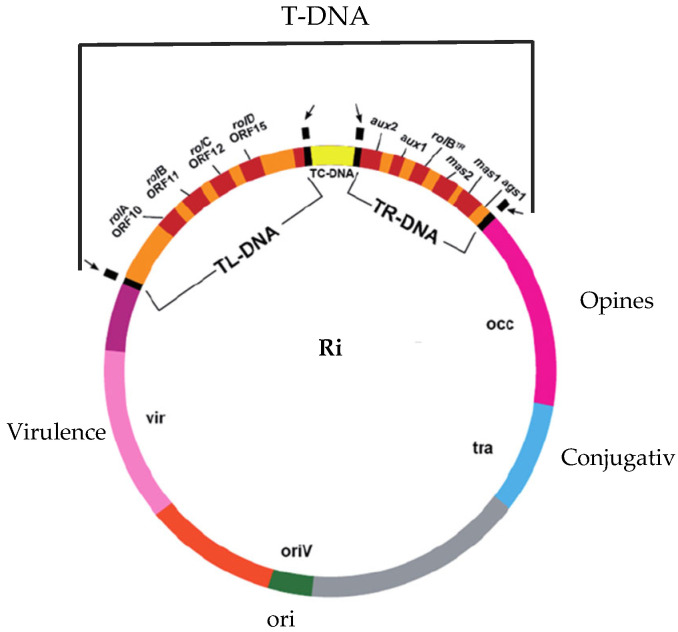
DNA plasmide of *R. rhizogenes* bacteria.

For molecular analysis of the incorporation of bacterial T-DNA in the transformed hairy root clones, PCR detection of genes from the left sequence of bacterial T-DNA (LT-DNA): *rol*A, *rol*B, *rol*C, and *rol*D, as well as from the right sequence of bacterial T-DNA (RT-DNA): *aux*2, *aux*1, *rol*B^TR^, *mas*2, *mas*1, and *ags*1, were performed. The localization of examined genes on *R. rhizogenes* plasmidic DNA is shown in Figure 2 [20].

**Figure 3:**
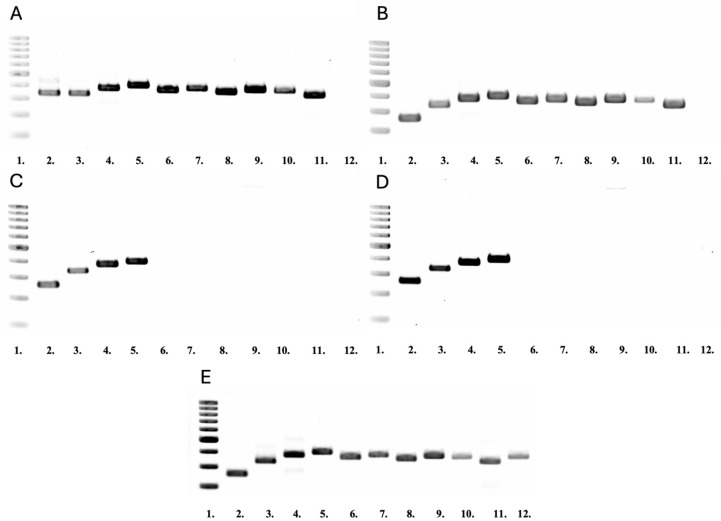
PCR analysis of the DNA from transformed hairy root cultures of *Reynoutria japonica*: (**A**). Clone RJ 30, (**B**). Clone RJ 9, (**C**). Clone RJ 10, (**D**). Clone RJ 11, and (**E**). from *Rhizobium rhizogenes* A4 (lanes 2–12). GeneRuler TM 100 pb Plus DNA ladder (lane 1). Amplified fragments of *rol*A (263 bp, lane 2); *rol*B (337 bp, lane 3); *rol*C (382 bp, lane 4); *rol*D (401 bp, lane 5); *aux*2 (363 bp, lane 6); *aux*1 (379 bp, lane 7); *rol*B^TR^ (358 bp, lane 8); *mas*2 (381 bp, lane 9); *mas*1 (373 bp, lane 10); *ags*1 (343 bp, lane 11); and *vir*G (371 bp; lane 12) genes.

The presence of whole LT-DNA (genes *rol*A, *rol*B, *rol*C, and *rol*D) and RT-DNA (genes *aux*2, *aux*1, *rol*B^TR^, *mas*2, *mas*1, and *ags*1) was detected in clones RJ 30 and RJ 9 (Figure 3A,B). Clones RJ 10 and RJ 11 contained genes only from LT-DNA (Figure 3C,D).

For confirmation of *R. rhizogenes* elimination from the tissue cultures of the transformed *R. japonica* clones, PCR for the *vir*G gene was also performed. This gene is present in the Ri plasmid (Figure 3E) but beyond the transferred T-DNA, and it cannot be incorporated into the plant genome. *Vir*G was not found in the transformed hairy root clones (Figure 3A–D).

### 2.3. Growth Parameters

**Figure 4:**
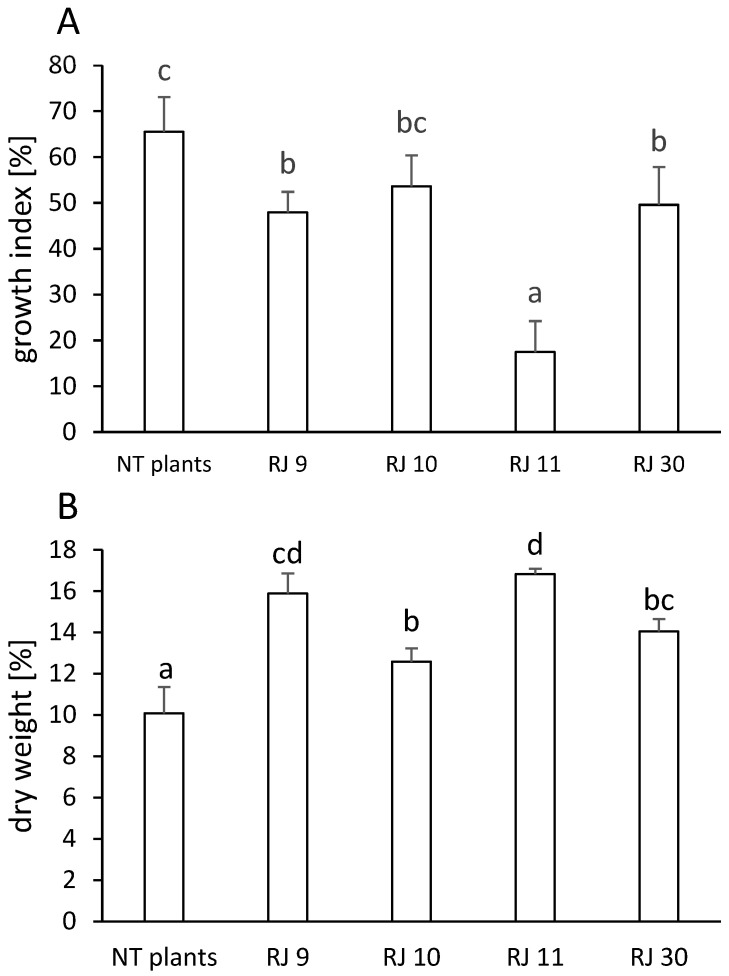
(**A**). Growth index [%] of non-transformed (NT plants) and hairy root cultures of *Reynoutria japonica* (clones: RJ 9, RJ 10, RJ 11, and RJ 30). (**B**). Dry weight content [%] of non-transformed (NT plants) and hairy root cultures of *R. japonica* (clones RJ 9, RJ 10, RJ 11, and RJ 30). Different letters indicate statistical significance of means acc. one-way ANOVA, post hoc Tuckey test at *p* < 0.05.

After 5 weeks of cultivating, the stabilized transformed hairy root cultures and non-transformed plants (NT plants) in liquid media with rotary shaking, growth index (GI), and dry weight content (DW) were evaluated. Estimation of growth parameters showed that clones RJ 9, RJ 11, and RJ 30 had a lower biomass accumulation than NT plants by 27, 72, and 27%, respectively (Figure 4A). The increased DW accumulation was shown for clones RJ 11 and 9, and then for clones RJ 30 and 10, it is higher than in NT plants by 67, 58, 39, and 25%, respectively (Figure 4B).

### 2.4. Accumulation of Phenolic Acids, Flawan-3-ols, and Flavonoids

The phenolic acid concentration in the transformed hairy root clones, shoots (NT shoot), and roots (NT root) of the NT plants were evaluated. The sum of phenolic acids was highest in the NT shoot, whereas the dominant derivative was caftaric acid also most accumulated in the NT shoot. The phenolic acid accumulation decreased from 7.5- to 12.9-fold in the transformed clones compared to the NT shoots. Protocatechuic acid was found in all transformed clones and NT roots, where its accumulation was the highest. Both the NT shoot and NT root accumulated more chlorogenic acid than the transformed hairy root clones. Analysis of caffeic acid showed that clone RJ 10 had the most of this metabolite, while clones RJ 9 and 30 did not synthesize it. The highest level of ferulic acid was found in the NT shoot (Table 1).

The chromatographic analysis also allowed for the estimation of flawan-3-ols content. This study revealed that transformation increased flawan-3-ols content in each hairy root clone compared to organs of non-transformed plants. The highest sum of flawan-3-ols was found in clone RJ 10. The increment in the sum of flawan-3-ols of transformed clones obtained a 2.6- and 1.8-fold increase compared to the NT shoot and root, respectively. The leading catechin derivative in transformed hairy roots was epicatechin, the accumulation of which was the highest in clone RJ 10 and RJ 11 obtaining a 4.4- and 4.2-fold increase in comparison to the NT shoot. Clones RJ 10, RJ 11, and RJ 30 had increased levels of epigallocatechin gallate compared to the NT shoot and NT root, while in clone RJ 9, it was not detected. The only flawan-3-ols with a high accumulation in NT shoot was epigallocatechin, whose accumulation was at the same level only in clone RJ 9 (Table 2).

This study allowed also for the estimation of the content of flavonoids not belonging to the flawan-3-ols derivatives. The highest sum of flavonoids was detected in the NT shoot. Nevertheless, clones RJ 10 and RJ 11 had increased 3.4- and 1.5-fold in the accumulation of flavonoids compared to NT roots, respectively. Isoquercetin was found only in organs of non-transformed plants, having the highest amount in NT shoot. Similarly, avicularin, quercetin, and trifolin synthesis were the highest in NT shoots. However, transformation led to an increased accumulation of avicularin in clones RJ 10 and RJ 11, as well as quercetin in clone RJ 10, in comparison to NT roots (Table 3).

### 2.5. Productivity of Phenolic Compounds

**Figure 5:**
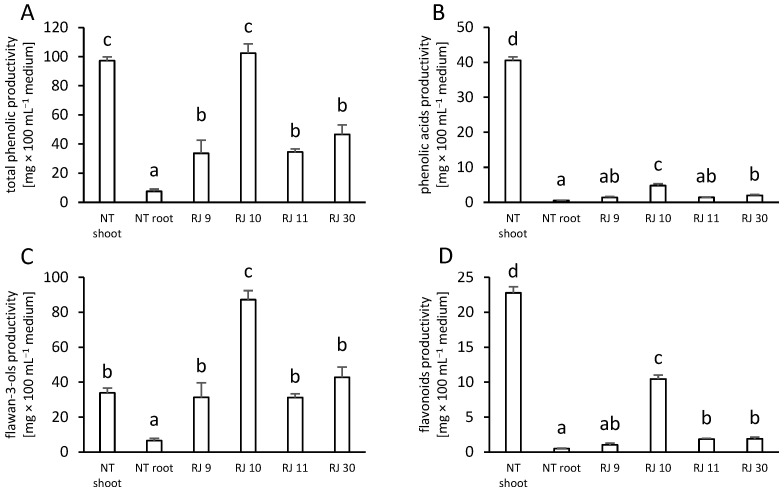
Productivity of (**A**). total phenolics, (**B**). phenolic acids, (**C**). flawan-3-ols, and (**D**). flavonoids [mg × 100 mL^−1^ medium] in non-transformed shoots (NT shoot), roots (NT root), and transformed hairy root cultures (clones: RJ 9, RJ 10, RJ 11, and RJ 30) of *Reynoutria japonica*. Different letters indicate statistical significance of means acc. one-way ANOVA, post hoc Tuckey test at *p* < 0.05, DW—dry weight.

An important parameter of the effectivity of phenolic metabolite production in tissue cultures is productivity. In our study, transformation affects total phenolic productivity in hairy root clones. Of these, clone RJ 10 had the most increased parameter, at the same level as NT shoots. The transformation led to higher total phenolic productivity in the remaining clones compared to NT roots (Figure 5A).

The highest phenolic acids productivity was found in NT shoots, while transformation increased this parameter in clones RJ 10 and RJ 30 beside the NT root (Figure 5B).

Flawan-3-ols productivity obtained a 2.6- and 13.4-fold increase in clone RJ 10 against the NT shoot and root, respectively. Other transformed clones were characterized by higher flawan-3-ols productivity than the NT root (Figure 5C).

Flavonoid productivity’s highest level was in the NT shoot, but transformation increased this variable in clones RJ 10, RJ 11, and RJ 30 compared to NT roots (Figure 5D).

### 2.6. Antioxidant Activity

**Figure 6:**
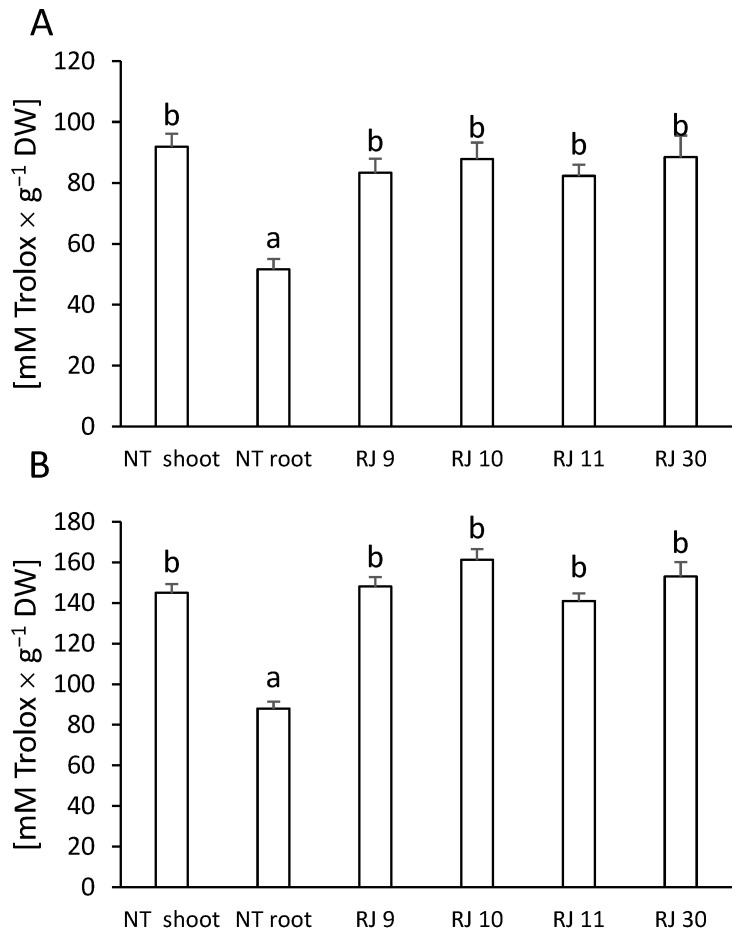
(**A**). DPPH radical scavenging activity [mM Trolox × g^−1^ DW] of non-transformed shoots (NT shoot) and roots (NT root) and hairy root cultures of *Reynoutria japonica* (clones: RJ 9, RJ 10, RJ 11, and RJ 30). (**B**). Antioxidant capacity [mM Trolox × g^−1^ DW] of non-transformed shoots (NT shoot) and roots (NT root) and hairy root cultures of *R. japonica* (clones: RJ 9, RJ 10, RJ 11, and RJ 30). Different letters indicate statistical significance of means acc. one-way ANOVA, post hoc Tuckey test at *p* < 0.05, DW—dry weight.

To evaluate the potential biological activity of the extracts derived from the examined tissues, two analyses measuring the reducing potential of the secondary metabolites contained in the extracts were used. Analysis of the results showed that transformation increased the ability of the extracts from each hairy root clone to scavenge the DPPH radical and reduce the copper ions compare to NT roots (Figure 6A,B).

### 2.7. Bactericidal Properties

Extracts from non-transformed and transformed tissues were tested for their ability to inhibit the growth (MIC) and kill (MBC) of three strains of *Staphylococcus aureus* bacteria. All samples tested, from both non-transformed organs and transformed hairy root clones, showed high antibacterial potential. The NT root showed around 80, 75, and 82% higher bactericidal properties (MBC value) against the reference, antibiotic-sensitive, and antibiotic-resistant bacteria strains, respectively, than the NT shoot. The transformation resulted in an increase in antimicrobial activity of clones RJ 10 and RJ 11 against the reference bacteria strain and RJ 10 against the antibiotic-sensitive bacteria strain compared to the NT root. The transformed clones were characterized by decreased bactericidal properties compared to NT roots against the antibiotic-resistant *S. aureus* strain (Table 4).

## 3. Discussion

Genetic transformation of medical plants with the use of wild strains of *R. rhizogenes* bacteria was proven to be a valuable biotechnology tool for the generation of hairy root or teratomas (transformed shoots) cultures with rapid and constant growth, a stable phenotype over extended periods, and an enhanced accumulation of biologically active secondary metabolites [21]. The presented study focused on the transformation of Japanese knotweed plants. Notably, for the first time, establishment of *R. japonica* transformed hairy root cultures was reported. The success of genetic transformation depends on several factors, among others: explant type and physiological state of tissue [1], selection of *R. rhizogenes* strain [22], cell division ability [23], and level of plant recalcitrance [24]. The effectiveness and efficiency of the transformation of medicinal plants are usually low due to the secondary metabolites present in their tissues, which may act against vector bacteria [25]. In this study, only the *R. rhizogenes* A4 strain proved to be effective in carrying out the transformation of *R. japonica,* contrary to our previous study about the transformation of another medical plant *Dionaea muscipula,* where this strain turned out to be ineffective [22]. Also, Ref. [3] showed that *R. rhizogenes* A4 had the lowest infection frequency during the transformation of *Cucumis anguria* plants. On the other hand, *R. rhizogenes* A4 turned out to be suitable for the transformation of *Leonotis nepetifolia* [26] or *Salvia viridis* [23]. The efficiency of the transformation process of *R. japonica* reached 64%. Additionally, Ref. [27] showed that *Brassica rapa* transformation efficiency was dependent on explant origin. Efficiency was 89% for leaves while only 11% for hypocotyls. *Momordica charantis* transformation efficiency with leaf explants was 91.5% [28], and Ref. [29] showed that the efficiency for the transformation of *Polygonum multiflorum* with leaf explants was 60%, while for petioles was 0%. The values of this parameter vary depending on the plant species, the explant selected for inoculation, and the bacterial strain used.

To confirm transformation at the molecular level, it is essential to ascertain the presence of bacterial genes within the plant genome. So far, most authors of studies on transformed plants have evaluated the presence of one or two potential bacterial oncogenes in plant DNA [3,30,31,32,33]. In recent years, a few studies have been published that postulate a variety of functions for bacterial oncogenes following their integration into the plant genome [5,6,34]. The elucidation of the fundamental mechanisms underlying bacterial oncogene action represents a crucial objective in the advancement of transformation as a biotechnological instrument. The process of transformation utilizing bacteria as vectors is inherently random, and the integration of genes from bacterial T-DNA into plant DNA is unpredictable. The location and number of copies of the integrated genes are also uncertain [35]. Accordingly, we undertook an investigation to ascertain the presence of all potential bacterial oncogenes within the genome of each of the examined clones. This approach enabled not only the confirmation of the transformation at the molecular level but also the molecular characterization of the transformants. Studies have shown that two transformed clones, RJ 9 and RJ 30, contained all the genes found in the bacterial T-DNA in their genome. This indicates that the right and left T-DNA sequences have been integrated into the genome of these clones (Figure 3A,B). Clones RJ 10 and RJ 11 possessed only the left bacterial T-DNA sequence as evidenced by the presence of all genes located on it and the absence of genes from the right sequence (Figure 3C,D). Given that the *rol* family genes (mainly *rol*B and *rol*C) are regarded as inducers of secondary metabolism in plants [4], the majority of studies on hairy root cultures focus on verifying their presence within the plant genome [36,37]. Nevertheless, a comprehensive molecular characterization of transformants enables their use in studies on the functions and actions of bacterial oncogenes in the plant genome.

The incorporation of bacterial T-DNA causes the development of hairy root syndrome in plants, which is characterized by rapid and stable biomass growth. This is related to changes in the auxin metabolism caused by bacterial oncogenes [38]. In this research, the selected hairy root clones were cultivated constantly in the five-week sub-cultures. Observations of the transformants showed similarity in the morphology of the clones (Figure 1), while analysis of the growth index (GI) and dry weight (DW) content revealed differences in terms of their growth level (Figure 4A,B). Clone RJ 11 demonstrated a diminished level of biomass production in comparison to other clones. At the same time, clones RJ 11 and RJ 9 exhibited elevated levels of dry weight accumulation compared to RJ 10 and RJ 30. Similar observations were made by [23] for transformed *S. viridis* clones. Each of them was characterized by a different biomass efficiency. Additionally, Ref. [31] showed that the transformed hairy roots of *C. anguria* plants grew faster than the roots of NT plants. Similarly, transformed hairy roots obtained from two chicory species, *Cichorium intybus* and *C. endivia*, were characterized by an increased growth index compared to NT plants [39]. Our study revealed that transformed Japanese knotweed clones had a lower growth index compared to NT plants. Nevertheless, they exhibited efficient biomass growth. Moreover, all transformed clones accumulated more dry mass, similar to the transformed *Aristolochia manshuriensis* hairy roots in the study of [40].

It has been revealed that the expression of bacterial oncogenes in the plant genome can evoke a stress response. One of the effects of this stress response is the increased synthesis of secondary metabolites [41]. The main inducers of secondary metabolism are the *rol*B and *rol*C genes [4]. Therefore, bacterial oncogenes can act as endogenous elicitors of secondary metabolites in plant tissues [42]. Increased production of phenolic compounds was reported in transformed hairy roots by [3,28,43], as well as in teratomas by [22,36] for *Momordica charantia*, *C. anguria*, *Dittrichia viscosa*, *Drosera capensis*, and *D. muscipula*, respectively. In this study, qualitative and quantitative phytochemical analysis using DAD-HPLC allowed for the identification and quantification of five phenolic acids derivatives and nine flavonoids, four of which were flawan-3-ols (catechin derivatives) in the examined tissues. Furthermore, the results were used to calculate the productivity of total phenolic acids, flawan-3-ols, and flavonoids (Figure 5). The studies have indicated that the genetic transformation of Japanese knotweed plants using wild strains of *R. rhizogenes* bacteria did not result in an increased accumulation of most of the phenolic acid derivatives tested in relation to NT roots. Notably, caffeic acid was not synthesized by clones RJ 9 and RJ 30, while its accumulation in clones RJ 10 and RJ 11 increased in comparison to NT roots (Table 1). Additionally, Ref. [2] showed that transformed hairy roots of *Salvia bulleyana* accumulated more phenolic acids than NT intact plants. Hairy roots of *C. anguria* had increased levels of gallic and chlorogenic acids [31], while *Ligularia fischeri* hairy roots had an increased accumulation of total phenolic acids [33]. Importantly, in cultures of the hairy root *R. japonica*, the transformation altered the metabolism of flawan-3-ols. Naturally, this group of flavonoids occurs in both the shoots and roots of NT plants. The studies showed that the presence of bacterial oncogenes in the plant genome led to an increase in the synthesis and accumulation of catechin, epicatechin, and epigallocatechin gallate. Similarly, in the transformed hairy roots of *M. dionica*, the level of catechin was higher than in NT roots [30]. Probably, the action of bacterial oncogenes may affect the catechin synthesis pathway. Transformation may also result in changes in the qualitative composition of secondary metabolites [21]. Clone RJ 9 inhibited the synthesis of epigallocatechin (Table 2). Transformation affected also the qualitative and quantitative composition of flavonoids. Isoquercetin was not accumulated in transformed clones, although it occurred in the NT shoot and NT root. As with phenolic acids, NT shoots naturally contained more isoquercetin, avicularin, quercetin, trifolin, and apigenin than the NT root. However, transformation resulted in increased synthesis of apigenin in clones RJ 10 and RJ 11, quercetin in clone RJ 10, apigenin in clones RJ 10, RJ 11, and RJ 30, and the sum of flavonoids in clone RJ 10 compared to the NT root (Table 3). Similarly, Ref. [44] reported increased levels of flavonoids in the hairy roots of *Salvia plebeia.* On the other hand, Ref. [30] showed a decrease in myricetin accumulation in transformed *M. dioica.* Nevertheless, increased total flavonoid content was reported for many transformed species [28,33,43,44]. The obtained results clearly indicate that the synthesis of phenolic metabolites in transformed hairy roots is dependent on physiological changes induced by bacterial oncogenes in individual clones.

An important parameter for assessing the efficiency of secondary metabolite production in transformed plant tissues may be the productivity. This parameter quantifies the amount of specific metabolite produced in relation to the biomass accumulation and input, which is a culture medium. In the study by [36], tearatomas of *D. capensis* plants had an increased productivity of phenolic derivatives. Also, teratomas of *D. muscipula* with *rol*B oncogene were characterized by an increased productivity of phenolic metabolites compared to NT plants [22]. In this research, the total phenolic productivity was much higher in NT shoot, than in NT root. Nevertheless, transformed hairy root cultures achieved higher productivity of total phenolics than NT root. Transformation exerted the most pronounced effect on productivity increase in clone RJ 10, whose productivity has reached the NT shoot level (Figure 5A). Moreover, clones RJ 10 and RJ 30 exhibited higher productivity of phenolic acids, than NT roots (Figure 5B). All the transformed clones tested demonstrated higher flawan-3-ols productivity than NT roots. The RJ 10 clone displayed the most pronounced flawan-3-ols productivity among all the tested objects (Figure 5C). The transformation process resulted in an increase in flavonoid productivity in RJ 10, RJ 11, and RJ 30 clones when compared to NT roots (Figure 5D). Our previous studies have demonstrated the impact of different tissue culture techniques on the production of phenolic compounds in medical plants [45,46].

One of the valuable indicators of the biological activity of extracts is their reducing potential, which reflects antioxidant activity [47]. Polyphenolic compounds are known for their protective properties against free radicals, both in plant physiology and as health-promoting compounds [48,49]. Consequently, this research included two analyses: DPPH and CUPRAC, which allowed for determining the antioxidant activity of the extracts from NT and transformed plants. The results demonstrated that the antioxidant effect of NT shoot extracts was more pronounced than that of NT root extracts. The genetic transformation resulted in an enhanced reducing potential of extracts from transformed clones compared to NT roots (Figure 6A,B). It was reported that transformed hairy roots may exhibit stronger antioxidant properties than NT roots. Additionally, Ref. [31] constated an elevated radical scavenging activity of *C. anguria*-transformed plants. Similar findings were described by [30,32,40], and [43]. In plants rich in phenolic compounds, Ref. [47] showed that one of the key groups that determine antioxidant potential may be phenolic acids. In our studies, NT shoots, richest in phenolic acids, have an increased antioxidant potential compared to NT roots. On the other hand, transformed clones, much poorer in phenolic acids than NT shoots, were characterized by antioxidant activity at the same level. We assume that, in the case of transformed hairy root cultures, flavonoids, particularly catechins, play a significant role in scavenging free radicals.

Medicinal plant species rich in phenolic compounds constitute a reservoir of biologically active substances that may be used in the future as a support/substitute for antibiotic therapy in infections with antibiotic-resistant pathogens [8]. It has been shown that some groups of polyphenols have a high bactericidal potential while not causing bacterial resistance [11]. Therefore, extracts from non-transformed and transformed plants of *R. japonica* were tested for their antibacterial properties. Studies have revealed that NT roots display a greater bactericidal potential than NT shoots. In addition, genetic transformation led to increased antibacterial properties of extracts from clones RJ 10 and RJ 11 in relation to the reference strain of bacteria and from clone RJ 10 in relation to the strain sensitive to antibiotics. Interestingly, all transformed clones were characterized by a reduced bactericidal potential in relation to NT root against antibiotic-resistant strains. Nevertheless, the bactericidal properties of transformed hairy roots were stronger for antibiotic-resistant bacteria in relation to NT shoots. Transformation of *C. anguria* increased antibacterial activity against *Escherichia coli* but had no effect on *S. aureus.* On the other hand, Ref. [33] showed that the transformation of *L. fischeria* increased the bactericidal properties of extracts for Gram-positive and Gram-negative pathogens. Gram-negative pathogens are often more resistant to plant secondary metabolites. This is due to the additional lipopolysaccharide layer present on their cell wall [22,47]. Further research is required to elucidate the antibacterial potential of secondary metabolites present in transformed Japanese knotweed plants.

## 4. Materials and Methods

### 4.1. Chemicals

Murashige & Skoog Medium with vitamins and claforan antibiotic = 98% were produced by Duchefa (Haarlem, The Netherlands). Sucrose = 99.8%, copper(II)chloride dihydrate (CuCl_2_) p.a., ammonium acetate (NH4Ac) p.a., methanol GR grade, and ethanol = 96% were purchased from Chempur (Piekary Śląskie, Poland). CTAB = 99% and neocuproine ≥ 99% was produced by Pol-Aura (Morąg, Poland). L-ascorbic acid ≥ 99%, DPPH radical, 2-(N-Morpholino)ethanesulfonic acid (MES) hydrate ≥ 99%, agar–agar, acetosyringone ≥ 98%, acetic acid (CH_3_COOH) glacial for chromatography, Trolox ≥ 98%, caftaric acid ≥ 97%, protocatechuic acid ≥ 95%, chlorogenic acid ≥ 95%, germanycaffeic acid = 98%, ferulic acid = 99%, epigallocatechi = 95%, (+)-catechin ≥ 96%, epigallocatechin gallate = 95%, epicatechin = 98%, isoquercetin ≥ 90%, trifolin ≥ 97%, avicularin ≥ 97%, quercitri ≥ 98%, and apigenin ≥ 95% were acquired from Sigma-Aldrich Co. (Hamburg, Germany). Carbenicillin antibiotic ≥ 88% was produced by Carl Roth GmbH (Karlsruhe, Germany). Sterilizing syringe filters 0.22 μm, Milex^®^GP Millipore was produced by Merck (Darmstadt, Germany). Mueller–Hinton broth (CA-MHB) medium was produced by Beckton Dickinson (Warszawa, Poland). Trypticase Soy Agar (TSA) was prepared based on chemicals from POCH S.A. (Gliwice, Poland).

### 4.2. Plant Material and Bacteria Strain Used for Transformation

*R. japonica* plants were cultivated in in vitro conditions according to [19]. Whole plant cultures were propagated on ½ Murashige and Skoog (MS) medium [50] with 3% of sucrose, 150 mg × L^−1^ L-ascorbic acid, 0.1% 2-(N-Morpholino)ethanesulfonic acid (MES) hydrate, and pH 5.8 (adjusted before autoclaving) with 0.75% of agar. Conditions included temperature of 20 ± 1 °C, fluorescence light of 80 μmol × m^−2^ × s^−1^ photosynthetic photon flux density (PPFD), and a photoperiod of 16 h/8 h light/dark cycle. Plants were sub-cultured in 4-week intervals. For the transformation, 15 developed and healthy plants were used.

Wild *R. rhizogenes* strains A4 (ATCC 31798), LBA 9402 (NCPPB 1855), and ATCC 15834 were grown according to [22] on yeast extract beef (YEB) agar medium [51] with 200 μM of acetosyringone at 26 °C in the dark. For plant transformation, 48 h bacterial cultures were used.

### 4.3. Transformation of R. japonica Plants

A total of 50 leaves of *R. japonica* were used as an explant for a transformation. Inoculation was performed with preparation needle, according to [36]. After inoculation, leaves were sub-cultured to ½ MS medium supplemented with 3% sucrose and 0.75% agar with pH 5.8 and grown for 3 days in the dark. Next, co-cultures were transferred to ½ MS medium supplemented with antibiotics claforan and carbenicillin (500 mg × L^−1^ each) to eliminate *R. rhizogenes*. After 4 weeks of cultivation in the dark, new transformed hairy roots of *R. japonica* were excised and placed in fresh liquid ½ MS medium with rotary shaking (120 rpm), containing the same antibiotic concentrations listed above, and grown in the dark for 4 weeks. After 5 sub-cultures (each passage reduced the antibiotic dose by 100 mg × L^−1^), axenic cultures were established from a single hairy root of transgenic tissue. Next, transformed clones were sub-cultured on fresh ½ MS media without antibiotic and growth regulators. Transformed clones of *R. japonica* hair roots were propagated for 8 weeks in liquid media with rotary shaking. During this time, observations of plant morphology, growth rate, and preliminary screening for phenolic compound quantity in comparison to non-transformed plants were performed. Based on these observations, four transformed hairy root clones, RJ 9, RJ 10, RJ 11, and RJ 30, were chosen for further analysis.

Transformed cultures were also tested for the presence of live *R. rhizogenes* found in tissue. Transformed shoots were homogenized, and the obtained suspensions were plated on YEB agar medium and grown for 5 days at 26 °C in the dark.

### 4.4. Molecular Characterization of Transformed Hairy Root Cultures

To confirm the transformation on molecular level and check which genes from bacterial T-DNA were incorporated into plant genomic DNA, the PCR reaction was performed. Total genomic DNA from transformed hairy root clones, RJ 9, RJ 10, RJ 11, and RJ 30, as well as from non-transformed plants was extracted using the CTAB method by [52]. This method is suitable for obtaining good-quality DNA free from secondary metabolites. As a positive control in PCR, plasmid DNA from bacteria cells was used. A culture of 24 h old *R. rhizogenes* (OD_600_ 4.0) was extracted using alkaline lysis as described by [53]. Primers for amplification of TL-DNA and TR-DNA region genes were designed based on the DNA sequence of the pRi plasmid from *Rhizobium rhizogenes* (GenBank accession number CP044124.1) using Clone Manager 9 Professional Edition software and Oligo Primer Analysis Software version 7.60. The primers were designed to ensure optimal DNA amplification conditions. Their optimal length was 20–24 bp, the annealing temperature for primer pairs ranged from 55 to 60 °C, and the amplicon length ranged from 263 to 401 bp. Oligonucleotide primers for the PCR detection of target genes are listed in Table 5:

### 4.5. Determination of Biometric Parameters

To estimate the growth of examined plants, transformed hairy roots and non-transformed plants were harvested after 5 weeks of cultivation and weighed immediately. A growth index (GI) was calculated according to the formula: GI [%] = (FW_2_ − FW_1_)/FW_2_ × 100, where FW_1_ was the fresh weight of plants at the beginning of this experiment, and FW_2_ was the final fresh weight. To determine dry weight (DW) accumulation, plants were freeze-dried for 24 h and weighed. DW content in plant tissue was calculated according to the formula: DW [%] = DW_2_ × 100/FW_2_, where DW_2_ was dry weight after freeze-drying. Freeze-dried plant tissue was homogenized and stored at −20 °C for further analysis.

### 4.6. Determination of Phenolic Compound Content

#### 4.6.1. Extraction Procedure

Plant extracts were prepared according to the procedure described by [45], with modifications. A total of 100 mg of homogenized freeze-dried tissue powder was weighed separately for shoots and roots of non-transformed plants and transformed hairy root clones. The tissue samples were extracted twice with 6 mL of 80% methanol by sonication in an ultrasonic bath (POLSONIC 2, Warszawa, Poland) for 30 min, 21 ± 2 °C. Sample for each research object was prepared in 3 biological repetitions (n = 3). The obtained extracts were centrifuged (15,000 rpm) and then filtered through sterilizing syringe filters (0.22 μm, Milex^®^GP, Millipore, Darmstadt, Germany) prior to HPLC analysis to the 1.5 mL vials as well as to 2 mL for screw-capped, airtight test tubes for spectrophotometric analysis.

#### 4.6.2. Phytochemical Analysis

To estimate the accumulation of phenolic compounds in plant tissue, high-performance liquid chromatography with a diode array detector (DAD-HPLC) was used. The quantitative analyses of selected secondary metabolites in the extracts were conducted with a validated method, using an apparatus of Merck-Hitachi, LaChrom Elite (Darmstadt, Germany) with a DAD L-2455 detector and on a Purospher RP-18 (250 × 4 mm; 5 μm, Merck, Germany) column according to former established and validated method according to [54,55]. The flow rate was 1 mL × min^−1^, the temperature was set to 25 °C, and the injection volume was 10 μL. The detection wavelength was set to 254 nm. The mobile phase consisted of A—methanol, 0.5% acetic acid in ratio of 1:4, and B—methanol (*v*/*v*). The gradient program was as follows: 0–20 min, 0% B; 20–35 min, 0–20% B; 35–45 min, 20–30% B; 45–55 min, 30–40% B; 55–60 min, 40–50% B; 60–65 min, 50–75% B; and 65–70 min, 75–100% B, with a hold time of 15 min. Identification was performed by comparison to the retention times and UV spectra of phenolic compounds: caftaric acid, protocatechuic acid, chlorogenic acid, caffeic acid, ferulic acid, epigallocatechin, catechin, epigallocatechin gallate, epicatechin, isoquercetin, trifolin, avicularin, quercitrin, and apigenin. For the regression equation and retention time see Supplementary Materials. The quantification was performed based on the calibration curves method. Samples were prepared and analyzed in five replications. The results were expressed in mg × 100 g^−1^ DW.

### 4.7. Determination of Phenolic Compounds Productivity

The productivity of phenolic compounds (PROD), total phenolic, phenolic acids, flawan-3-ols, and flavonoids in shoots and roots of non-transformed plants and transformed hairy root clones, was calculated according to the following formula: PROD [mg × 100 mL^−1^ of medium] = A × B/C, where A was concentration of the sum of phenolic compounds/phenolic acids/flawan-3-ols/flavonoids in plant tissue after 5 weeks of growth per 100 g DW, B was the quantity of g DW in one flask, and C was 100 mL of medium in the flask.

### 4.8. Determination of Antioxidant Activity

Estimation of antioxidant activity of extracts was performed using two methods: cupric-ion-reducing antioxidant capacity assay (CUPRAC) [56] and radical scavenging capacity assay with 2,2-diphenyl-1-picrylhydrazyl free radical (DPPH) [57] with modifications of [45,49]. The same methanolic extracts used for HPLC studies were used for spectrophotometric measurements. Extracts were diluted with ultra-pure water, 16 times for the CUPRAC assay and 4 times for the DPPH assay.

For the CUPRAC assay, 1 mL of 10 mM CuCl_2_, 1 mL of 7.5 mM neocuproine in 96% ethanol, and 1 mL of 1 M NH_4_Ac buffer (pH 7.0) were mixed with 0.3 mL of the diluted methanolic extract and 0.8 mL of water. The absorbance was measured at 450 nm after 5 min. The results were expressed as mmol Trolox per 1 g of DW.

The mixture for the estimation of DPPH radical scavenging capacity contained 2.8 mL of 0.1 mM DPPH solution in 96% ethanol and 0.2 mL of diluted methanolic extract. The DPPH absorbance was detected after 5 min, at 517 nm. The results were expressed as mmol Trolox per 1 g of DW.

### 4.9. Antibacterial Activity

To evaluate the bactericidal properties of extracts, minimal inhibitory concentration (MIC) and minimal bactericidal concentration (MBC) were determined with broth microdilution method for each tested plant extract [58,59]. Parameters were evaluated against human–pathogenic bacteria *Staphylococcus aureus.* Examined strains were characterized by different sensitivity to antibiotics: ATCC 25922 was reference strain, while 1521 [60] was sensitive to antibiotics, and 614k was strain resistant to more than one group of commercial antibiotics [60]. All bacteria strains were obtained from the IFB UG & MUG Poland. The bacteria were cultivated on cation-adjusted Mueller–Hinton broth (CA-MHB) medium (overnight, 37 °C). Freeze-dried plant tissue (100 mg) was extracted in 6 mL of 80% methanol [46]. Extracts were evaporated and resuspended in methanol before application into wells of the 96-well plate. To remove toxic methanol, extracts were evaporated in the wells. The residues were suspended in 100 μL of CA-BHI medium, and 10 μL aliquots of bacterial suspension in CA-BHI (10^5^ CFU × mL^−1^) were added to each well. Plates were incubated overnight at 37 °C. To establish the MBC value, 100 μL from each well that showed no visible growth of bacteria were plated out on a Trypticase Soy Agar (TSA) plate for 24 h of incubation at 37 °C. The MBC was defined as the lowest concentration of the extract that reduced the inoculum by 99.9% within 24 h.

### 4.10. Statistic Analysis

One-way analysis of variance (ANOVA) was used to determine significant differences between means (Tukey test at *p* < 0.05 level). In case of two means (isoquercetin concentration), difference was indicated using Student’s *t*-test at *p* < 0.05. STATISTICA 12.0 (StatSoft Inc., Tulsa, OK, USA) was used to carry out statistical analyses.

## 5. Conclusions

The present study demonstrated the first successful genetic transformation of *R. japonica*. The incorporation of bacterial oncogenes into the genomic DNA of the Japanese knotweed plant resulted in the endogenous elicitation of catechin derivatives. This suggests that transformed Japanese knotweed clones may constitute an efficient and stable platform for the production of these compounds. Furthermore, it has also been indicated that Japanese knotweed tissues, both untransformed and transformed, exhibit a broad spectrum of biological activity and may serve as a source of secondary metabolites with a high bactericidal potential. We hypothesize that the high antioxidative potential of extracts derived from transformed clones is predominantly associated with an increased flawan-3-ols accumulation. Moreover, results have shown that hairy root cultures of *R. japonica* may be a source of secondary metabolites with bactericidal properties against antibiotic-resistant *S. aureus*.

## Figures and Tables

**Table 1:** Accumulation of phenolic acids [mg × 100 g^−1^ DW] in non-transformed shoots (NT shoot), roots (NT root), and transformed hairy root cultures (RJ 9, RJ 10, RJ 11, and RJ 30) of *Reynoutria japonica.* Different letters indicate statistical significance of means acc. one-way ANOVA, post hoc Tuckey test at *p* < 0.05, SD—standard deviation, DW—dry weight, nd—not detected. Statistical analysis performed for each of the tested parameters (the content of a given compound and the sum) separately.

The Object of Examination	Caftaric Acid	Protocatechuic Acid	Chlorogenic Acid	Caffeic Acid	Ferulic Acid	Sum of Phenolic Acids
	mg × 100 g^−1^ DW [±SD]					
NT shoot	461.1 ± 33.9 b	nd	51.8 ± 1.9 c	1.8 ± 0.4 bc	31.8 ± 3.1 c	546.4 ± 28.7 b
NT root	10.8 ± 0.8 a	7.7 ± 0.3 b	15.8 ± 0.6 b	0.9 ± 0.1 ab	14.2 ± 0.3 ab	49.4 ± 1.2 a
RJ 9	17.9 ± 0.4 a	2.5 ± 0.1 a	11.1 ± 1.1 a	nd	10.7 ± 1.4 a	42.3 ± 2.8 a
RJ 10	37.9 ± 0.3 a	1.9 ± 0.2 a	10.6 ± 0.1 a	8.4 ± 1.0 d	13.7 ± 4.0 ab	72.5 ± 5.0 a
RJ 11	24.4 ± 0.6 a	2.2 ± 0.4 a	9.2 ± 0.8 a	2.6 ± 0.3 c	14.3 ± 0.7 ab	52.6 ± 1.8 a
RJ 30	19.2 ± 0.8 a	2.3 ± 0.1 a	10.5 ± 0.4 a	nd	16.8 ± 0.9 b	48.8 ± 0.5 a

**Table 2:** Accumulation of flawan-3-ols [mg × 100 g^−1^ DW] in non-transformed shoots (NT shoot), roots (NT root), and transformed hairy root cultures (clones: RJ 9, RJ 10, RJ 11, and RJ 30) of *Reynoutria japonica.* Different letters indicate statistical significance of means acc. one-way ANOVA, post hoc Tuckey test at *p* < 0.05, SD—standard deviation, DW—dry weight, nd—not detected. Statistical analysis performed for each of the tested parameters (the content of a given compound and the sum) separately.

The Object of Examination	Epigallocatechin	Catechin	Epigallocatechin Gallate	Epicatechin	Sum of Flawan-3-ols
	mg × 100 g^−1^ DW [±SD]				
NT shoot	136.6 ± 16.7 c	124.4 ± 15.6 a	9.7 ± 1.5 b	184.6 ± 21.8 a	455.3 ± 25.9 a
NT root	57.6 ± 9.6 a	120.6 ± 2.3 a	3.1 ± 0.5 a	457.6 ± 53.3 b	638.9 ± 50.2 b
RJ 9	142.9 ± 3.4 c	317.8 ± 14.9 c	nd	495.0 ± 18.8 bc	955.7 ± 32.9 c
RJ 10	62.0 ± 1.7 a	397.9 ± 9.8 d	55.8 ± 0.9 d	807.7 ± 11.9 d	1323.4 ± 21.4 f
RJ 11	72.2 ± 1.6 a	275.9 ± 7.3 b	46.8 ± 0.5 c	781.6 ± 54.3 d	1176.3 ± 61.0 e
RJ 30	94.9 ± 1.6 b	334.4 ± 1.9 c	61.7 ± 1.8 e	581.5 ± 10.5 c	1072.5 ± 10.3 d

**Table 3:** Accumulation of flavonoids [mg × 100 g^−1^ DW] in non-transformed shoots (NT shoot), roots (NT root), and transformed hairy root cultures (clones: RJ 9, RJ 10, RJ 11, and RJ 30) of *Reynoutria japonica*. Different letters indicate statistical significance of means acc. one-way ANOVA, post hoc Tuckey test at *p* < 0.05. For isoquercetin, statistical difference was indicated using Student’s *t*-test at *p* < 0.05. SD—standard deviation, DW—dry weight, nd—not detected. Statistical analysis performed for each of the tested parameters (the content of a given compound and the sum) separately.

The Object of Examination	Isoquercetin	Avicularin	Quercetin	Trifolin	Apigenin	Sum of Flavonoids
	mg × 100 g^−1^ DW [±SD]					
NT shoot	25.9 ± 0.8 *	10.8 ± 0.2 d	185.9 ± 2.8 b	10.6 ± 0.4 d	73.1 ± 1.5 e	306.2 ± 1.6 e
NT root	7.3 ± 0.8	1.1 ± 0.2 a	2.9 ± 0.1 a	4.0 ± 0.7 c	31.9 ± 0.4 b	47.0 ± 1.5 b
RJ 9	nd	1.0 ± 0.2 a	1.3 ± 0.1 a	0.6 ± 0.1 a	28.6 ± 0.5 a	31.6 ± 0.6 a
RJ 10	nd	2.5 ± 0.1 c	6.7 ± 0.1 b	2.3 ± 0.1 b	147.4 ± 0.7 f	158.8 ± 0.6 d
RJ 11	nd	1.9 ± 0.2 b	2.7 ± 0.4 a	1.6 ± 0.5 ab	63.3 ± 0.5 d	69.6 ± 1.0 c
RJ 30	nd	1.4 ± 0.1 a	2.2 ± 0.1 a	0.7 ± 0.1 a	43.2 ± 0.7 c	47.5 ± 0.7 b

**Table 4:** Minimum inhibitory concentration (MIC) and minimum bactericidal concentration (MBC) [mg DW × mL^−1^] of non-transformed shoots (NT shoot), roots (NT roots), and transformed hairy root cultures (clones: RJ 9, RJ 10, RJ 11, and RJ 30) of *Reynoutri japonica* against *Staphylococcus aureus* strains. DW—dry weight.

	*Staphylococcus aureus* ATCC 25922 (Reference Strain)		*Staphylococcus aureus* 1521 (Antibiotic-Sensitive Strain)		*Staphylococcus aureus* 614k (Antibiotic-Resistant Strain)	
	MIC	MBC	MIC	MBC	MIC	MBC
	mg DW × mL^−1^					
NT shoot	3.33	8.33	3.33	6.67	3.33	9.17
NT root	1.25	1.67	1.67	1.67	1.67	1.67
RJ 9	1.25	1.67	1.25	1.67	1.67	5.00
RJ 10	1.04	1.25	1.25	1.46	1.67	4.17
RJ 11	1.04	1.46	1.25	1.67	1.67	4.17
RJ 30	1.25	1.88	1.25	1.67	1.67	5.00

**Table 5:** Sequences of primers used in PCR reactions.

Primer Name	Sequence [5′-3′]
*rol*A-F	CCC AGA CCT TCG GAG TAT TAT CG
*rol*A-R	CCG TAG GTT TGT TTC GAA ATG CG
*rol*B-F	GAG TCG CAG GGT TAG GTC TGG C
*rol*B-R	CGT GCT GGC GAC AAC GAT TCA AC
*rol*C-F	GAG GAT GTG ACA AGC AGC GAT G
*rol*C-R	CCA TCT GCT CAT TCA GCT TGA TG
*rol*D-F	CTA TAT ATC ATC TGC AAC TGA GC
*rol*D-R	CCA GTT CCC TAC TAT AAA TCT TG
*aux*2-F	GGA GCT GTT GGG AAA AGA ATT G
*aux*2-R	CTT AGC AGC CGA GAC GAT TAT C
*aux*1-F	GTA GAC GAT GTT ACG GTG TAT G
*aux*1-R	GCT GTA GAT GCC CTA ACT TC
*rol*B^TR^-F	GAT AAG AAA GGG GCA CAG GAC C
*rol*B^TR^-R	CCT TAC GCG AAA AGT ATG CTA CC
*mas*2-F	CGA TGA CCT GAA AGC TTA TCT CG
*mas*2-R	CAC TGC TTC CGG CTC TTA TTT C
*mas*1-F	CTC TGC TTT GAC ATC GAC GAG G
*mas*1-R	CAA TCA GGT CTT CGG CGA TGG
*ags*1-F	CTA CGC TTG ATT ACT GCC ACT G
*ags*1-R	GAG AGT TGT TAG CTG AAG ATG AGG
*vir*G-F	CTT AAT TTG GGT CGC GAA GAT GGG
*vir*G-R	GAT AGA ACG TCG CGC GGC TTC

## Data Availability

The data presented in this study are available on request from the corresponding author.

## Supplementary Materials

The following supporting information can be downloaded at: https://www.mdpi.com/article/10.3390/ijms26010362/s1.
