# Supplementary material for: Reynoutria japonica Houtt. Transformed Hairy Root Cultures as an Effective Platform for Producing Phenolic Compounds with Strong Bactericidal Properties
Source: Int J Mol Sci. 2025 Jan 3;26(1):362. doi: 10.3390/ijms26010362 (PMC11720400; doi:10.3390/ijms26010362)
Supplement: Supplementary file 1 [file ijms-26-00362-s001.zip › ijms-3375377-supplementary.pdf]

Regression equation and retention time for analyzed phenolic derivatives:

1) caftaric acid (y=1E+08x-13409; R^2=0.9903; t_R=5.52 min),
2) protocatechuic acid (y=1E+078x+159880; R^2=0.9991; t_R=6.98 min),
3) chlorogenic acid (y=4E+07x-45223; R^2=0.9997; t_R=11.67 min),
4) caffeic acid (y=6E+07x-169512; R^2=0.9996; t_R=16.96 min),
5) ferulic acid (y=6E+07x+30835; R^2=0.9983; t_R=36.91 min),
6) epigallocatechin (y=3E+06x-30719; R^2=0.9999; t_R=7.53 min),
7) catechin (y=6E+06x+54243; R^2=0.9991; t_R=8.51 min),
8) epigallocatechin gallate (y=3E+07x-148672; R^2=1.0; t_R=15.43 min),
9) epicatechin (y=6E+06x-48481; R^2=0.9994; t_R=19.03 min),
10) isoquercetin (y=9E+07x+1113000; R^2=0.9999; t_R=45.42 min),
11) trifolin (y=8E+07x+583711; R^2=0.9994; t_R=49.25 min)
12) avicularin (y=1E+08x-1E+09; R^2=0.9995; t_R=49.40 min),
13) quercitrin (y=7E+07x-813339; R^2=0.9979; t_R=49.90 min),
14) apigenin (y=6E+07x-1E+06; R^2=0.9999; t_R=64.65 min).
